# Treatment with the Proteasome Inhibitor MG132 during the End of Oocyte Maturation Improves Oocyte Competence for Development after Fertilization in Cattle

**DOI:** 10.1371/journal.pone.0048613

**Published:** 2012-11-07

**Authors:** Jinyoung You, Eunsong Lee, Luciano Bonilla, Jasmine Francis, Jin Koh, Jeremy Block, Sixue Chen, Peter J. Hansen

**Affiliations:** 1 College of Veterinary Medicine, Kangwon National University, Chunchon, Korea; 2 Department of Animal Sciences and D.H. Barron Reproductive and Perinatal Biology Research Program, University of Florida, Gainesville, Florida, United States of America; 3 Interdisciplinary Center for Biotechnology Research, University of Florida, Gainesville, Florida, United States of America; 4 Ovatech LLC, Gainesville, Florida, United States of America; 5 Dept. of Biology, University of Florida, Gainesville, Florida, United States of America; National Cancer Institute, United States of America

## Abstract

Maturation of the oocyte involves nuclear and cytoplasmic changes that include post-translational processing of proteins. The objective was to investigate whether inhibition of proteasomes during maturation would alter competence of the bovine oocyte for fertilization and subsequent development. Cumulus-oocyte complexes were cultured in the presence or absence of the proteasomal inhibitor MG132 from either 0–6 h or 16–22 h after initiation of maturation. Treatment with MG132 early in maturation prevented progression to meiosis II and reduced fertilization rate and the proportion of oocytes and cleaved embryos that became blastocysts. Conversely, treatment with MG132 late in maturation improved the percentage of oocytes and cleaved embryos that became blastocysts without affecting nuclear maturation or fertilization rate. Optimal results with MG132 were achieved at a concentration of 10 µM – effects were generally not observed at lower or higher concentrations. Using proteomic analysis, it was found that MG132 at the end of maturation increased relative expression of 6 proteins and decreased relative expression of 23. Among those increased by MG132 that are potentially important for oocyte competence are GAPDH, involved in glycolysis, TUBA1C, needed for organellar movement, and two proteins involved in protein folding (P4HB and HYOU1). MG132 decreased amounts of several proteins that exert anti-apoptotic actions including ASNS, HSP90B1, PDIA3 and VCP. Another protein decreased by MG132, CDK5, can lead to apoptosis if aberrantly activated and one protein increased by MG132, P4HB, is anti-apoptotic. Finally, the pregnancy rate of cows receiving embryos produced from oocytes treated with MG132 from 16–22 h of maturation was similar to that for control embryos, suggesting that use of MG132 for production of embryos in vitro does not cause a substantial decrease in embryo quality.

## Introduction

The proteasome, a multisubunit proteolytic complex involved in degradation of ubiquitinated proteins, plays a crucial role in assuring completion of meiosis and formation of a developmentally-competent embryo. Early in maturation, completion of meiosis I requires inactivation of maturation promoting factor (MPF) through a process mediated by proteasomal cleavage of ubiquitinated cyclin B1 [1]. Other aspects of oocyte function during maturation are also under control of the proteasome. In mice, for example, the proteasome is required for the initiation and maintenance of translation of mRNA for the RNA binding protein *SLBP* [2]. Abundance of another protein involved in RNA processing, CPEB, is under negative regulation by proteasomes in *Xenopus* [3]. In addition, cumulus cells encasing the oocyte require proteasomal activity for optimal function as indicated by negative effects of the proteasomal inhibitor MG132 on progesterone production and expression of genes involved in expansion of the extracellular matrix [4]. This peptide aldehyde, N-(benzyloxycarbonyl)leucinylleucinylleucinal, functions as a substrate analog and transition-state inhibitor of the chymotrypsin-like activity of the proteasome [5].

Late in the process of oocyte maturation, the proteasome may contribute to a reduction in the functional properties of the oocyte. Treatment with MG132 reduced the effect of in vitro aging on oocyte competence in the mouse [6]. Furthermore, treatment of oocytes with MG132 late in maturation increased abundance of specific transcripts and improved developmental competence of parthenogenetically-activated oocytes in the pig [7].

If inhibition of the ubiquitin-proteasome pathway late in maturation improves oocyte competence, it may be possible to improve the success rate of assisted reproductive technologies that utilize in vitro matured oocytes. The purpose of the present series of experiments was to test the hypothesis that treatment of bovine oocytes with MG132 at the end of maturation would improve developmental competence of the oocytes and resultant embryos while addition of MG132 at the beginning of maturation would reduce oocyte competence. An additional goal was to assess specific proteins whose relative abundance in the oocyte was altered by MG132 late in maturation with the goal of identifying candidate molecules responsible for actions of MG132 on oocyte competence.

## Materials and Methods

Use of animals was approved by the University of Florida Institutional Animal Care and Use Committee.

### Culture Media

Chemicals were obtained from Sigma-Aldrich Chemical Company (St. Louis, MO, USA) or Fisher (Pittsburgh, PA, USA) unless otherwise stated. The base medium for oocyte maturation (OMM) was Tissue Culture Medium-199 (TCM-199; Invitrogen, Carlsbad, CA) with Earle’s salts supplemented with 10% (v/v) bovine steer serum containing 2 U/ml heparin (Pel-Freez, Rogers, AR, USA), 2 μg/ml estradiol 17-β, 20 μg/ml bovine follicle stimulating hormone (Folltropin-V; Bioniche Life Sciences, London, ON, Canada), 22 μg/ml sodium citrate, 50 μg/ml gentamicin sulfate and 1 mM glutamine. Oocyte collection medium (OCM) was TCM-199 medium with Hank’s salts (Cellgro, Mediatech, Manassas, VA, USA) supplemented with100 U/ml penicillin-G, 0.1 mg/ml streptomycin, 1 mM glutamine and 2% (v/v) bovine steer serum containing 2 U/ml heparin. HEPES-Tyrodes albumin lactate pyruvate solution (TALP) was prepared as described previously [8]. The fertilization medium was in vitro fertilization (IVF)-TALP [8]. Percoll was from GE Healthcare Bio-Sciences AB (Uppsala, Sweden). Frozen semen from bulls of various breeds was donated by Southeastern Semen Services (Wellborn, FL, USA). The embryo culture medium was SOF-BE1 [9] or, for one experiment, a proprietary culture medium called BBH7 from Minitube (Verona, WI, USA). Hoechst 33342 was purchased from Sigma-Aldrich. The MG132 was purchased from Sigma-Aldrich.

### Oocyte Collection and In Vitro Maturation (IVM)

Bovine ovaries were obtained from various breeds at a local abattoir (Central Packing, Center Hill, Florida) and transported to the laboratory. The owner provided permission to use the ovaries for experimental purposes. Cumulus-oocyte complexes (COCs) were collected by slicing superficial follicles (2–10 mm in diameter) with a scalpel blade and washing the ovaries into a beaker containing OCM. The COCs were harvested using a capillary pipette and washed three times in OCM. Groups of 10 were placed into 50 μl drops of OMM covered with mineral oil. The COCs were matured for 22 h at 38.5°C in a humidified atmosphere of 5% (v/v) CO_2_, with MG132 treatment applied according to the experimental design. The MG132 was dissolved in dimethyl sulfoxide (DMSO) and was added to maturation drops so that the final concentration of DMSO was not greater than 0.5% (v/v). The control oocytes were cultured with medium supplemented with a similar amount of DMSO during IVM as for oocytes treated with MG132.

### In Vitro Fertilization and Culture

After maturation, all COCs were washed twice in HEPES-TALP and once in IVF-TALP and then transferred in groups of 30–50 oocytes to 425 μl of fertilization medium in wells of a 5-well dish (Minitube, Verona, WI, USA). Oocytes were fertilized with a pool of frozen-thawed sperm from three bulls purified by Percoll gradient centrifugation [8]; different pools were used in each replicate. Oocytes were fertilized (day of fertilization termed Day 0) by adding 30 μl of Percoll-purified spermatozoa (final concentration in fertilization dish  = 1×10^6^ sperm cells/ml in IVF-TALP and 20 μl PHE (0.5 mM penicillamine, 0.25 mM hypotaurine and 25 μΜ epinephrine in 0.9% (w/v) NaCl). After 8 h at 38.5°C and 5% (v/v) CO_2_ in humidified air, putative zygotes were removed from fertilization wells, denuded of cumulus cells by vortexing in hyaluronidase (10,000 U/ml in 600 μl HEPES-TALP) for 4 min, washed in HEPES-TALP, and placed in groups of 20 to 35 putative zygotes in 50 μl microdrops of SOF-BE1 medium overlaid with mineral oil at 38.5°C in a humidified atmosphere of 5% (v/v) CO_2_, 5% O_2_ and the balance nitrogen. Cleavage and blastocyst formation were evaluated on Days 3 and 8 after IVF, respectively.

### Examination of Nuclear Status of Oocytes after IVM

At 16 h and 22 h of IVM, COCs were transferred into HEPES-TALP containing 0.3% (w/v) hyaluronidase and then vortexed for 5 min to remove cumulus cells. Denuded oocytes were stained with 5 μg/ml Hoechst 33342 in 10 mM PBS (10 mM PO_4_, 0.9% (w/v) NaCl) containing 1% (w/v) polyvinylpyrrolidone (PBS-PVP) for 1 h at room temperature. Then, 10–15 oocytes were mounted on glass slides with a small amount of anti-fade solution (Life Technologies, Grand Island, NY, USA and covered with a cover slip. Oocytes were examined using an Axioplan 2 epifluorescence microscope (Zeiss, Göttingen, Germany) with blue filter (excitation wavelength = 365/12 nm; emission wavelengths = 395–750 nm). Each oocyte was classified according to stage of nuclear maturation as germinal vesicle (GV), germinal vesicle break down (GVBD), pre-metaphase I-metaphase I (MI), anaphase I (AI)-telophase I (TI) and metaphase II (MII).

### Examination of Pronuclear Status of Oocytes after IVF

Inseminated oocytes were harvested from culture drops of SOF-BE1 at 18 h after fertilization, mounted on glass slides and nuclei visualized using Hoechst 33342 as described above for oocytes. Oocytes were classified as non-penetrated if the nucleus was at MI or MII without the presence of a sperm head or male pronucleus. An oocyte was classified as fertilized if one swollen sperm head or male pronucleus was detected inside the oocyte. Oocytes having more than one swollen sperm head or male pronuclei were classified as polyspermy.

### Blastocyst Cell Number

Blastocysts were fixed for 1 h at room temperature in 4% (w/v) paraformaldehyde dissolved in PBS. After washing in PBS-PVP, embryos were incubated with 1 µg/ml Hoescht 33342 dissolved in PBS-PVP. Embryos were washed in PBS-PVP, placed on a microscope slide and number of nuclei counted using a Zeiss Axioplan 2 epifluorescence microscope (Zeiss, Göttingen, Germany).

### Experiments on Oocyte Maturation, Fertilization and Development (Experiments 1–6)

The concentration-dependent effects of MG132 added at the end of oocyte maturation on embryonic development were tested in Experiments 1 and 2. COCs were matured in OMM that was supplemented with 0, 1, 5, 10 μM MG132 (Experiment 1) or 0, 10, 20 or 30 μM MG132 (Experiment 2) from 16 h to 22 h after initiation of maturation. Treatment was achieved by washing COCs after 16 h of maturation and placing them in fresh medium containing MG132 or vehicle. Endpoints were cleavage rate at day 3 after insemination, the proportion of oocytes and cleaved embryos that became blastocysts at Day 8, and blastocyst cell number. The experiments were replicated six times with 20–50 COCs per treatment for each replicate (Experiment 1) and four times with 20–30 COCs per treatment for each replicate (Experiment 2).

Experiment 3 was conducted to determine whether timing of MG132 treatment altered effects of the inhibitor on embryonic development. COCs were untreated or treated with 10 μM MG132 at two times [0–6 h of maturation (during the initiation of maturation) or 16–22 h of maturation (at the end of maturation)] using a 2×2 factorial arrangement of treatments. The COCs were placed in appropriate treatments at 0 h (vehicle or MG132), washed at 6 h, placed in fresh medium without MG132, washed at 16 h of maturation, and placed in fresh medium with appropriate treatment. Thus, some cultures received vehicle at 0–6 h and 16–22 h, some received MG132 from 0–6 h and 16–22 h, some received MG132 from 0–6 h and vehicle from 16–22 h, and some received vehicle from 0–6 h and MG132 from 16–22 h. Endpoints were cleavage rate at day 3 after insemination and the proportion of oocytes and cleaved embryos that became blastocysts at Day 8. The experiment was replicated six times with 20–50 COCs per treatment for each replicate.

Experiments 4–6 were conducted to determine effects of MG132 on oocyte nuclear maturation (Experiments 4 and 5) and fertilization rate (Experiment 6). For Experiment 4, COCs were treated with vehicle or 10 μM MG132 for the first 6 h of maturation and nuclear maturation was examined at 16 h after initiation of maturation. The experiment was replicated three times with 20–35 COCs per treatment for each replicate. For Experiments 5 and 6, COCs were untreated or treated with 10 μM MG132 at two times (0–6 h of maturation, 16–22 h of maturation, or at both times) using a 2×2 factorial arrangement of treatments and procedures as described for Experiment 3. The endpoints were nuclear maturation at 22 h of maturation (Experiment 5) or sperm penetration at 18 h after exposure to sperm (Experiment 6). Experiment 5 was replicated three times with 20–35 COCs per treatment for each replicate. Experiment 6 was replicated four times with 20–50 COCs per treatment for each replicate.

Data were analyzed statistically as follows. For each replicate, percentage data (for example, percentage of oocytes that cleaved and percentage of cleaved embryos that became blastocysts) were calculated for all oocytes or embryos within the same treatment. Thus, the group of oocytes treated alike within each replicate was the experimental unit. Statistical analyses were performed using the Statistical Analysis System (version 9.2; SAS Institute Inc., Cary, NC, USA). Data were analyzed using the General Linear Models procedure. For main effects with more than 1 degree of freedom, the pdiff mean separation procedure was used when main effects or interactions differed at *P<*0.10. Percentage data were arcsine-transformed prior to analysis to maintain homogeneity of variance. Results are expressed as least-squares means ± standard error of the mean (SEM) of the untransformed data.

### Effect of MG132 on the Oocyte Proteome (Experiment 7)

Oocytes were matured as described above. After 16 h of maturation, COCs were placed in fresh medium containing 10 µM MG132 or vehicle. The COCs were denuded after 22 h of maturation by vortexing after treatment with hyaluronidase. Those oocytes in which a polar body was evident by light microscopy were retained and processed for protein extraction. The zona pellucida was removed by treatment for 5 min with 0.1% (w/v) protease from *Streptomyces griseus* followed by mechanical shearing.

Three biological replicates were included for both vehicle and MG132 groups. A biological replicate represented a pool of polar-body-extruded oocytes collected from several oocyte maturation procedures. The number of oocytes per pool was 225 for replicate 1, 225 for replicate 2 and 1000 for replicate 3. Oocytes were suspended in 10 mM KPO_4_, pH 7.4 containing 1 mg/ml polyvinyl alcohol and 1% (w/v) protease inhibitor cocktail (Sigma) and stored at −70°C until processing. Total protein was isolated from pooled oocytes and purified as described elsewhere [10]. The protein concentration was determined using the BCA® Protein Assay (Thermo, Rockford, IL, USA).

For each sample (regardless of the number of starting oocytes), 100 µg protein was dissolved in protein buffer [0.2% (w/v) sodium dodecyl sulfate, 8 M urea, and 10 mM Triton X-100). The samples were reduced, alkylated, trypsin-digested, and labeled following the manufacturer’s instructions for the iTRAQ Reagents 4-plex kit (AB Sciex Inc., Foster City, CA, USA). To verify the tag efficiency of the iTRAQ method, iTRAQ tags 114 and 115 were used to label control samples and tags 116 and 117 were used to label MG132 groups. Two iTRAQ procedures were conducted. In Set 1, one control and one MG132 sample were analyzed twice to determine technical replication. In Set 2, two biological replicates of each treatment were analyzed. Proteins were identified using an off-line 2D liquid chromatography-MS/MS method with strong cation exchange (SCX) chromatography as a first step to fractionate the oocyte proteome (Figure S1). The tryptic peptide mixtures were lyophilized, dissolved in SCX solvent A [25% (v/v) acetonitrile, 10 mM ammonium formate, and 0.1% (v/v) formic acid, pH 2.8], and fractionated using an Agilent HPLC system 1260 with a polysulfoethyl A column (2.1 × 100 mm, 5 µm, 300 Å; PolyLC, Columbia, MD, USA). Tryptic peptides were separated with a LC Packing C18 Pep Map HPLC column (Dionex, San Francisco, CA, USA), and a hybrid quadrupole-TOF QSTAR Elite MS/MS system (AB Sciex Inc., Framingham, MA, USA) was used for data acquisition.

The MS/MS data were processed by a thorough search considering biological modification and amino acid substitution against the National Center for Biotechnology Information non-redundant *Bos taurus* fasta database (83,655 entries) and uniprot *B. taurus* database (33,808 entries) under the Paragon™ algorithm [11] using ProteinPilot v.4.2 software (Applied Biosystems). After searching MS/MS spectra against these databases, results were combined into each group. Animal species, fixed modification of methylmethane thiosulfate-labeled cysteine, fixed iTRAQ modification of amine groups in the N-terminus and lysine, and variable iTRAQ modifications of tyrosine were considered. The ProteinPilot cutoff score was set to 1.3 (a confidence level of 95%), and the false discovery rate (FDR) was estimated by performing the search against concatenated databases containing both forward and reverse sequences (Table S1).

For protein quantification, we only considered MS/MS spectra that were unique to a particular protein and where the sum of the signal-to-noise ratio for all of the peak pairs was >9 (software default settings, AB Sciex Inc.). The accuracy of each protein ratio was calculated by the ProGroup analysis in the software to determine whether the protein is significantly differentially expressed [12]. To be identified as being significantly differentially expressed, a protein must have been quantified with at least three spectra, the fold change was >1.2 or <0.8, and the P value for vehicle vs MG132 was <0.05 as determined with Fisher’s combined probability test [13] (Fisher, 1948). The strength of the protein signal is referred to as relative expression because the total amount of protein analyzed was similar for MG132 and vehicle-treated oocytes. Differentially expressed proteins were analyzed for GO terms by Blast2GO [14] and, after conversion to official gene symbols, by the Database for Annotation, Visualization and Integrated Discovery [DAVID; (DAVID Bioinformatics Resources 6.7, http://david.abcc.ncifcrf.gov/)] [15]. For DAVID, genes were annotated using the bovine genome as a reference. In addition, functional properties of differentially-abundant proteins were determined by mining PUBMED using the Chilibot program (www.chilibot.net) [16].

### Pregnancy Rates after Transfer of Embryos Produced using MG132 During Oocyte Maturation (Experiment 8)

Embryos were produced in vitro using Holstein COCs that were collected from abattoir-derived ovaries (Central Packing, Center Hill, FL). Maturation was carried out using conditions similar to those for other experiments. At 16 h of maturation, COCs were washed and then placed in fresh medium containing 10 µM MG132 or vehicle. Fertilization was carried out for 8 h in SOF-IVF [17] using X-sorted semen from a single Holstein bull (Accelerated Genetics, Baraboo, WI, USA and Select Sires, Plain City, OH, USA). A total of 4 different bulls were used in the experiment. Sperm were purified before fertilization as described elsewhere [18]. The final sperm concentration in the fertilization well was 1×10^6^ sperm/ml. Following fertilization, presumptive zygotes were cultured in 50 μl microdrops of BBH7 culture medium overlaid with mineral oil in groups of 25–30 in a humidified atmosphere of 5% CO_2_ and 5% O_2_ (balance N_2_) at 38.5°C.

Grade 1 expanded blastocysts [19] were harvested at d 7 after insemination and vitrified using the open-pulled straw method as described elsewhere [18]. On the day of transfer, open-pulled straws were thawed and contents emptied into a 2-well plate (Agtech, Manhattan, KS, USA) filled with thaw medium [Tissue Culture Medium 199 with Hank’s salts and supplemented with 10% (v/v) fetal bovine serum and 50 µg/mL gentamicin] containing 0.33 M sucrose. Immediately afterwards, embryos were transferred to a fresh well of the same medium. Embryos were then loaded individually into 0.25 mL embryo transfer straws and transferred immediately thereafter (<5 min after thawing).

Embryos were transferred to lactating female Holsteins on four occasions between June 10, 2011 and August 19, 2011 at the University of Florida Dairy Unit (Hague, FL; 29.77904 N, 82.48001 W). Cows were housed in free-stall barns equipped with fans and sprinklers. They were fed a total mixed ration and milked two times per day. Cows were either first-service cows or cows that had previously been inseminated or received an embryo during that lactation and had been diagnosed non-pregnant. Cows were subjected to the Ovsynch-56 timed ovulation protocol [20]. Specifically, cows received 100 µg gonadotropin releasing hormone (GnRH), i.m., on d –10; 25 mg prostaglandin F_2α_ (PGF), i.m., on d –3; and 100 µg GnRH, i.m., at 56 h after PGF. For first-service cows only, the timed ovulation protocol was preceded by a presynchronization protocol (two injections of 25 mg PGF, i.m., 14 d apart), with the last injection 12 d before initiation of the Ovsynch-56 protocol.

Embryos were transferred on day 7 of the above-mentioned synchronization protocol to cows diagnosed by ultrasonography as having a corpus luteum on the scheduled day for embryo transfer. Each cow received a single embryo in the uterine horn ipsilateral to the corpus luteum. Cows were paired and randomly assigned within pair to receive an embryo produced with vehicle or MG132. Transfer was achieved transcervically and cows received an epidural block (5 mL 2% lidocaine, w/v) before transfer. Pregnancy was diagnosed by ultrasound at d 32 and by rectal palpation at d 46 and 71. A total of 24 embryos produced with vehicle and 30 embryos produced with MG132 were transferred.

Data on cleavage and blastocyst development were analyzed by least-squares analysis of variance as described for Experiments 1–6 (n = 10 replicates) while data on pregnancy rate were analyzed by chi-square analysis.

## Results

### Concentration-dependent Effect of MG132 from 16–22 h of Maturation on Subsequent Embryonic Development (Experiments 1 and 2)

In the first experiment, COCs were treated from 16 to 22 h of maturation with 0, 1, 5 or 10 μM MG132 (Table 1). The highest concentration of MG132 increased (*P*<0.05) the percentage of oocytes that cleaved (i.e., that were ≥2 cells) and the percentage of oocytes that became blastocysts. There was, however, no effect of 10 μM MG132 on the percentage of cleaved embryos that became blastocysts or on the number of cells per blastocyst. There were also no effects of lower concentrations of MG132 on any endpoint.

**Table 1:** Effects of MG132 (1–10 µM) added from 16–22 h of maturation on subsequent embryonic development (Experiment 1).^a^

MG132, µM	No. of oocytes	Percentage of oocytes developing to		Percentage of cleaved embryos developing to the blastocyst stage	No. of cells in blastocyst
		≥2-cell	Blastocyst		
0	241	74.5±1.3 ^b^	35.9±2.8 ^b^	48.6±3.4^ b^	146.5±1.7 ^b^
1	232	75.9±1.3 ^b^	32.9±2.7 ^b^	43.9±3.2^ b^	147.2±1.7 ^b^
5	224	75.6±1.3 ^b^	31.7±2.7 ^b^	42.7±3.2^ b^	146.6±1.7 ^b^
10	259	86.6±1.3 ^c^	49.8±2.7 ^c^	54.8±3.2^ b^	146.9±1.7^ b^

a Data are least-squares means ± SEM of values from six replicates.

b,c Values in the same column with different superscript letters are significantly different (*P*<0.05).

In Experiment 2, COCs were treated with 0, 10, 20 or 30 μM MG132 (Table 2). As shown in Experiment 1, treatment of COCs with 10 μM MG132 increased cleavage rate and the percentage of oocytes becoming blastocysts (*P*<0.05). In addition, the percentage of cleaved embryos becoming blastocysts was increased (P<0.05) by treatment with 10 μM MG132. As in Experiment 1, there was no effect of 10 μM MG132 on blastocyst cell number. Treatment with 20 μM MG132 increased (P<0.05) cleavage rate but did not affect other endpoints examined. Treatment with 30 μM MG132 had no effect on subsequent development.

**Table 2:** Effects of MG132 (10–30 µM) added from 16–22 h of maturation on subsequent embryonic development (Experiment 2).^a^

MG132, µM	No. of oocytes	Percentage of oocytes developing to		Percentage of cleaved embryos developing to the blastocyst stage	No. of cells in blastocyst
		≥2-cell	Blastocyst		
0	241	60.9±2.4 ^b^	21.3±1.6 ^b^	35.0±2.4^ b^	154.9±1.5 ^b^
10	232	74.3±2.3 ^c^	35.0±1.5 ^c^	46.8±2.3^ c^	155.4±1.5 ^b^
20	224	69.1±2.3 ^cd^	24.4±1.5 ^b^	34.9±2.3^ b^	154.2±1.5 ^b^
30	259	64.0±2.3 ^bd^	22.0±1.5 ^b^	34.2±2.3^ b^	153.5±1.5^ b^

a Data are least-squares means ± SEM of values from four replicates.

b,c,d Values in the same column with different superscript letters are significantly different (*P*<0.05).

### Actions of MG132 During the First Six or Last Six Hours of Maturation (Experiment 3)

Results of Experiments 1 and 2 indicated that the response of COCs to MG132 occurred over a narrow range and that optimal effects on maturation were achieved with 10 μM MG132. Consequently, subsequent experiments were performed with MG132 at this concentration. For Experiment 3, it was tested whether MG132 would affect maturation differently when added at 0–6 h of maturation, when proteasomes are necessary for completion of meiosis I, than when added at 16–22 h of maturation (Table 3). When added from 0–6 h, addition of MG132 reduced the proportion of oocytes that cleaved and the proportion of oocytes and cleaved embryos that became blastocysts (main effect of MG132, P<0.01 or less). Addition of MG132 from 16–22 of maturation increased (P<0.05) the percentage of oocytes undergoing cleavage. MG132 from 16–22 h also increased the percentage of oocytes and cleaved embryos developing to the blastocyst stage provided COCs were not also treated with MG132 from 0–6 h (interaction, P<0.05). Addition of MG132 from 16–22 h increased (P<0.02) blastocyst cell number slightly but differences were not detected using the pdiff mean separation test.

**Table 3:** Effect of treatment with 10 μM MG132 from 0–6 or 16–22 h of maturation on subsequent embryonic development (Experiment 3).^a^

MG132,0–6 h	MG132, 16–22 h		No. of oocytes	Percentage of oocytes developing to			Percentage of cleaved embryos developing to the blastocyst stage	No. of cells in blastocyst
				≥2-cell		Blastocyst		
No	No		164	58.8±2.3^b^		22.4±1.9^b^	38.0±2.4^b^	142.4±2.2^b^
No	Yes		143	68.4±2.3^c^		34.8±1.9^c^	49.3±2.4^c^	148.7±2.2^b^
Yes	No		161	34.5±2.3^d^		10.9±1.9^d^	29.9±2.4^bd^	141.3±2.5^b^
Yes	Yes		166	41.0±2.3^d^		10.2±1.9^d^	24.1±2.4^d^	144.1±2.2^b^
Probability of treatment effects^e^								
MG132, 0–6				0.001	0.01		0.01	N.S.
MG132, 16–22				0.05	N.S.		N.S.	P<0.02
Interaction				N.S.	0.05		0.05	N.S.

a Data are least-squares means ± SEM of values from six replicates.

b,c,d Values in the same column with different superscript letters are significantly different (*P*<0.05).

e N.S. = non-significant (P>0.10).

### Nuclear Maturation of Bovine Oocytes Treated with or without MG132 from 0–6 h after Maturation (Experiment 4) or 16–22 h after Maturation (Experiment 5)

It was hypothesized that MG132 treatment from 0–6 h of maturation reduced cleavage rate and the percentage of oocytes becoming blastocysts because it blocked progression through meiosis I. This hypothesis was examined in Experiment 4 (Table 4). Indeed, MG132 treatment from 0–6 h of maturation increased (P<0.05) the proportion of oocytes that were at metaphase I at 16 h after maturation and tended (P>0.10) to decrease the number of oocytes that were at metaphase II. A second experiment (Experiment 5) was conducted in which MG132 was added at either 0–6 or 16–22 h of maturation (Table 5). In general, treatment effects were not significant except that there was an interaction (P<0.09) affecting the percentage of oocytes at metaphase I. In particular, the percentage of oocytes at metaphase I was increased by treatment with MG132 at 0–6 h while treatment from 16–22 h increased the percentage of oocytes at MI when MG132 was not also added at 0–6 h. While not significant, MG132 treatment from 0–6 h also tended to reduce the percentage of oocytes that were at metaphase II.

**Table 4:** Effect of treatment with MG132 from 0–6 h of maturation on meiotic maturation at 16 h after initiation of maturation (Experiment 4).^a^

MG132, µM	No. of oocytes	Nuclear status, % b			
		GVBD	MI	Ana-Telo	MII
0	67	1.3±2.0 ^c^	34.8±5.0^ c^	14.7±2.7 ^c^	49.3±6.0^ c^
10	76	4.9±2.0 ^c^	53.4±5.0^ d^	11.0±2.7 ^c^	30.8±6.0^ c^

a Data are least-squares means ± SEM of values from three replicates.

b GVBD: germinal vesicle break down; MI: metaphase I; Ana-Telo: anaphase – telophase; MII: metaphase II.

c,d Values in the same column with different superscript letters are significantly different (*P*<0.05).

**Table 5:** Effect of treatment with 10 μM MG132 from 0–6 or 16–22 h of maturation on meiotic maturation at 22 h after initiation of maturation (Experiment 5).^a^

MG132, 0–6 h	MG132, 16–22 h	No. of oocytes	Nuclear status, % b				
			GVBD		MI	Ana-Telo	MII
No	No	91	0.9±1.2^c^		14.2±0.8^c^	0.0±2.9^c^	84.9±3.5^c^
No	Yes	79	0.0±1.2^c^		19.1±0.8^d^	6.0±2.9^c^	74.8±3.5^c^
Yes	No	69	0.0±1.2^c^		34.8±0.8^e^	3.2±2.9^c^	62.0±3.5^c^
Yes	Yes	69	1.6±1.2^c^		34.7±0.8^e^	3.6±2.9^c^	60.2±3.5^c^
Probability of treatment effects^f^							
MG132, 0–6				N.S.	N.S.	N.S.	N.S.
MG132, 16–22				N.S.	N.S.	N.S.	N.S.
Interaction				N.S.	P<0.09	N.S.	N.S.

a Data are least-squares means ± SEM of values from three replicates.

b GVBD: germinal vesicle break down; MI: metaphase I; Ana-Telo: anaphase – telophase; MII: metaphase II.

c,d,e Values in the same column with different superscript letters are significantly different (*P*<0.05 or, for).

f N.S. = non-significant (P>0.10).

### Fertilization Rates of Oocytes Treated with MG132 from 0–6 or 16–22 h of Maturation (Experiment 6)

Results are in Table 6. Addition of MG132 from 0–6 h of maturation reduced fertilization rate regardless of whether MG132 was also added at 16–22 h of maturation (P<0.05). There was no effect of MG132 from 16–22 h on fertilization rate. There was a tendency (P<0.07) for addition of MG132 from 0–6 h to decrease the percentage of oocytes with polyspermy.

**Table 6:** Effect of treatment with 10 μM MG132 from 0–6 or 16–22 h of maturation on fertilization rate (Experiment 6).^a^

MG132, 0–6 h		MG132, 16–22 h		No. of oocytes			Percentage ofoocytes fertilized	Percentagepolyspermy			
No		No		136			72.1±2.5^bcd^		12.1±2.9^b^		
No		Yes		138			81.7±2.5^c^		14.2±2.9^b^		
Yes		No		110			59.3±2.5^d^		8.1±2.9^b^		
Yes		Yes		120			57.8±2.5^d^		7.9±2.9^b^		
Probability of treatment effects^e^											
MG132, 0–6							P<0.05		P<0.07		
MG132, 16–22							N.S.		N.S.		
Interaction							N.S.		N.S.		

a Data are least-squares means ± SEM of values from four replicates.

e N.S. = non-significant (P>0.10).

### The Effect of MG132 Treatment on Protein Expression of Matured Oocytes (Experiment 7)

**Figure 1:**
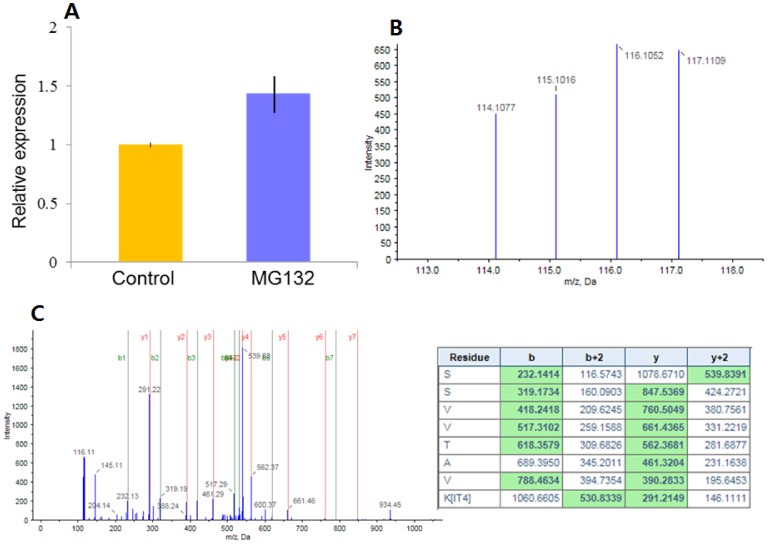
Expression levels and detection of peptide of Cullin-associated NEDD8-dissociated protein1 (CAND1). Panel A: Mean ± SEM of CAND1 expression for control and MG132-treated oocytes. There was a difference (P = 0.004) between treatments. Panel B: Reporter ion expression for the C peptide fragment of CAND1. 114 and 115 represent two separate biological replicates of control oocytes while 116 and 117 represent two separate biological replicates of MG132-treated oocytes. Panel C: b and y ions and amino acid sequence from one peptide fragment of CAND1.

Using iTRAQ labeling and the 2D LC-MSMS method, a total of 669 proteins was identified in matured oocytes with 653 having a reporter ion region. A list of these proteins and differences in relative amount between oocytes treated with MG132 and vehicle are shown in File S1. Relative expression of 7 distinct proteins increased in response to MG132 whereas relative expression of 24 distinct proteins was decreased (Table 7). Representative results for one differentially-expressed protein, CAND1, is shown in Figure 1, including mean ± SEM of CAND1 expression for control and MG132-treated oocytes (Figure 1A), example of reporter ion expression for the C peptide fragment of CAND1 from one iTRAQ procedure (Figure 1B), and an example of b and y ions and amino acid sequence from one peptide fragment of CAND1 (Figure 1C).

**Table 7:** Proteins whose abundance was altered by 10 µM MG132 from 16–22 h of maturation.

Accession number	Gene symbol		Name	Fold change^a^	P
gi|85682743	GAPDH		RecName: Full = Glyceraldehyde-3-phosphate dehydrogenase	3.01	0.027
gi|332634826	HYOU1		hypoxia up-regulated protein 1	2.30	0.014
tr|E1B748|E1B748_BOVIN			Uncharacterized protein	1.99	0.004
tr|A6H7J6|A6H7J6_BOVIN		P4HB	P4HB protein	1.72	0.027
sp|Q3ZCJ7|TBA1C_BOVIN		TUBA1C	Tubulin alpha-1C chain	1.60	0.018
sp|A7MBJ5|CAND1_BOVIN		CAND1	Cullin-associated NEDD8-dissociated protein 1	1.44	0.004
sp|Q29RZ0|THIL_BOVIN		ACAT1	Acetyl-CoA acetyltransferase, mitochondrial	1.36	0.016
gi|74355032		SLC25A5	Solute carrier family 25 (mitochondrial carrier; adenine nucleotide translocator), member 5	0.37	0.039
gi|78369310		STIP1	stress-induced-phosphoprotein 1	0.45	0.002
gi|296470781		UBA1	ubiquitin-activating enzyme E1	0.46	0.010
tr|F1MHP6|F1MHP6_BOVIN			Uncharacterized protein	0.48	0.013
sp|Q8SQH5|ADT2_BOVIN		ADT2	ADP/ATP translocase 2	0.48	0.021
tr|F1MF89|F1MF89_BOVIN			Uncharacterized protein (Fragment)	0.49	0.010
gi|296486956		ADSL	adenylosuccinate lyase	0.49	0.024
sp|Q3MHL4|SAHH_BOVIN		AHCY	Adenosylhomocysteinase	0.52	0.039
tr|F1N785|F1N785_BOVIN			Uncharacterized protein	0.53	0.044
tr|Q1JPA2|Q1JPA2_BOVIN		EEF1G	Eukaryotic translation elongation factor 1 gamma (Fragment)	0.53	0.001
gi|3336842		ALB	bovine serum albumin	0.54	0.030
gi|296475166		PDIA3	protein disulfide-isomerase A3 precursor	0.54	0.021
gi|296484906		PLAA	phospholipase A2-activating protein	0.56	0.043
gi|95767537		THOP1	thimet oligopeptidase 1	0.58	0.011
sp|Q02399|CDK5_BOVIN		CDK5	Cyclin-dependent kinase 5	0.59	0.021
tr|Q3T0K7|Q3T0K7_BOVIN		MFGE8	MFGE8 protein	0.63	0.021
sp|Q1LZA3|ASNS_BOVIN		ASNS	Asparagine synthetase [glutamine-hydrolyzing]	0.63	0.016
tr|F1MEN8|F1MEN8_BOVIN			Uncharacterized protein	0.64	0.014
gi|7545448		MGP57/53	MGP57/53 glycoprotein antigen	0.64	0.021
tr|A5D7E8|A5D7E8_BOVIN		PDIA3	PDIA3 protein	0.66	0.005
sp|Q3ZBT1|TERA_BOVIN		VCP	Transitional endoplasmic reticulum ATPase	0.70	0.016
gi|75775556		HSP90B1	Tumor rejection antigen (gp96) 1	0.71	0.002
tr|E1BBC4|E1BBC4_BOVIN			Uncharacterized protein	0.73	0.021
tr|Q2KIV8|Q2KIV8_BOVIN		GSTM3	Glutathione S-transferase mu 3 (Brain)	0.75	0.000

a MG132/vehicle.

Analysis of molecular function GO terms using DAVID revealed that six proteins (all lower in MG132 treated oocytes) were classified in the regulation of apoptosis term (HSP90B1, PDIA3, VCP, ALB, ASNS, CDK5), 5 in the macromolecule catabolic process term (HSP90B1, VCP, UBA1, and CDK5 lower for MG132 and CAND1 higher for MG132) and 5 in the proteolysis term (HSP90B1, VCP, UBA1, and THOP1 lower for MG132 and CAND1 higher for MG132). Other GO terms were synonymous to these terms or included fewer proteins that were affected by MG132.

To determine the degree to which the proteome of the bovine oocyte matches published oocyte proteomes, we evaluated whether a subset of randomly-chosen proteins (minimum of 2 peptides detected) in the present database was present in a database of proteins identified in mouse oocytes [21]. Of the 125 proteins examined, 73 (58%) were identified in the mouse.

### Pregnancy Rates after Transfer of Embryos Produced with MG132 (Experiment 8)

Treatment of oocytes with MG132 from 16–22 h of maturation increased (P<0.06) cleavage rate from 48.8% to 62.6% (SEM = 4.8%). While numerically greater, the effect of MG132 on percentage of oocytes becoming blastocysts was not significant (12.4% vs 19.2% for vehicle and MG132, SEM = 3.3%). Note that the reduced cleavage and blastocyst rates in this experiment reflect the use of X-sorted sperm for fertilization.

As shown in Table 8, there was no significant effect of MG132 on pregnancy rate at 32, 46 or 71 d of gestation.

**Table 8:** Effect of treatment with 10 µM MG132 from 16–22 h of maturation ability on the ability of the resultant blastocysts to establish pregnancy after transfer to recipient females.

MG132 (µM)	Pregnancy rate at various days of gestation, fraction and percentage		
	Day 32	Day 46	Day 71
0	9/24 (38%)	7/24 (29%)	6/20 (30%)
10	12/30 (40%)	12/30 (40%)	7/23 (30%)

## Discussion

Oocyte competence for nuclear maturation, fertilization and ability to support embryonic development was affected by addition of the proteasomal inhibitor MG132 during the maturation process. Actions of MG132 depended on the time of addition. Oocyte competence was improved when MG132 was added during the last 6 h of maturation (from 16–22 h of maturation) and reduced when added during the first 6 h of maturation.

It is well established that proteasomal activity is required for completion of meiosis I. Proteasomal cleavage of ubiquitinated cyclin B1 leads to the inactivation of MPF required for completion of meiosis I [1]. Inhibition of meiosis is likely a major cause for reduced oocyte competence caused by addition of MG132 from 0–6 h of maturation because MG132 treatment at this time tended to reduce the proportion of oocytes that reached MII at the end of maturation. Inhibition of other proteasome-mediated events early in maturation may also be involved in reduced oocyte competence. For example, in the pig, MG132 can affect cumulus cells by reducing progesterone production and expression of genes involved in expansion of the extracellular matrix [4].

The finding that treatment with MG132 late in maturation improves oocyte competence is consistent with other results showing beneficial effects of MG132 on aged mouse oocytes fertilized by intracytoplasmic sperm injection [6] and parthenogenetically activated pig oocytes [7]. Beneficial effects of MG132 on nuclear remodeling, transcript abundance and embryonic development have also been shown for embryos constructed by somatic cell nuclear cloning in mice [22], [23], rats [24], [25], goats [23] and pigs [7], [26], [27]. Unlike for addition from 0–6 h, MG132 added from 16–22 h did not improve oocyte competence by improving nuclear maturation because the percentage of oocytes that were MII at the end of maturation was not affected by MG132 later in maturation. Rather, some of the beneficial effect of MG132 from 16–22 h on the percentage of oocytes that became blastocysts was due to 1) increased cleavage rate through actions not involving fertilization rate and 2) increased competence of the fertilized oocyte to develop to the blastocyst stage. Indeed, the potential of a newly formed embryo to become a blastocyst was improved by addition of MG132 from 16–22 h in two of three experiments evaluated, as indicated by a significant improvement in the percentage of cleaved embryos that became blastocysts.

The mechanism by which MG132 late in maturation improves competence of the oocyte to support development is likely to involve arrest of processes mediated by proteasomes that ordinarily compromise the oocyte. One result is likely to be increased transcript abundance for genes required for embryonic development, as shown in the pig oocyte [7]. In the mouse, MG132 improved oocyte competence in aged oocytes but did not affect non-aged oocytes [6]. It might be that MG132 blocked proteasome-mediated degenerative changes in a portion of maturing oocytes of inferior quality caused by prolonged culture during maturation or other reasons.

Proteomic analysis was performed to determine possible targets of proteasomal cleavage whose relative expression was altered by MG132 treatment from 16–22 h. Such proteins might be involved in the beneficial effects of MG132 on oocyte competence and may be important molecules for determining the ability of an oocyte to complete the first cleavage division and support development of the embryo to the blastocyst stage. One limitation to the experimental approach was that less abundant proteins were less likely to be detected by mass spectrometry. Nonetheless, a total of 653 proteins could be analyzed for differences in amount between oocytes treated with vehicle or MG132. Surprisingly, there were a greater number of proteins whose relative expression was decreased by MG132 than there were proteins that were increased. Regulation of intracellular proteins in the presence of MG12 is complex. In HEK293T cells, MG132 can increase ubiquitination of some proteins and decrease ubiquitination of others [28]. Some proteins in the bovine oocyte increase in abundance during oocyte maturation whereas others decline in amount [29]. It is possible that inhibition of the proteasome by MG132 late in maturation protected some proteins from proteolysis, which in turn hastened or exaggerated the maturation-dependent decline in other oocyte proteins. Six of the proteins that were decreased by MG132 (ADSL, AHCY, CDK5, GSTM3, STIP1, and THOP1) and two that were increased by MG132 (CAND1 and GAPDH) are encoded for by transcripts that decrease during nuclear maturation of bovine oocytes [30]. Among the oocyte proteins regulated by the proteasome are proteins involved in RNA processing [2], [3] so inhibition of proteasomal activity with MG132 could affect stability and translation of a variety of mRNA.

There were 6 annotated proteins identified whose relative expression was increased by MG132 (ACAT1, CAND1, TUBACA1C, P4HB, HYOU1, and GAPDH). The increase in GAPDH may be a direct result of inhibition of the proteasome because intracellular amounts of GAPDH are regulated by ubiquitination [31], [32]. Another mechanism may be involved in regulation of CAND1 by MG132. This protein interferes with ubiquitin ligase activity [33]. Perhaps, inhibition of cleavage of ubiquitinated proteins leads to increased synthesis or decreased degradation of CAND1 through feedback mechanisms. Other proteins involved in the ubiquitin pathway were decreased by MG132, notably HSP90B1, THOP1, UBA1, and VCP.

None of the 6 annotated proteins increased by MG132 have been identified as a marker of oocyte competence. Nonetheless, an increase in amounts of these proteins could potentially affect oocyte competence. GAPDH, for example, catalyzes an important step in glycolysis. Glycolysis in the bovine oocyte is low and most pyruvate for the oocyte is supplied by the surrounding cumulus cells [34]. There is some evidence, though, that rate of glycolysis in the bovine oocyte is proportional to developmental competence [35]. Another protein increased by MG132 was TUBA1C. Tubulins are important for organelle movement in the oocyte and completion of meiosis [36], [37]. Two other upregulated proteins, P4HB and HYOU1, function in protein folding [38], [39].

The maturing oocyte is capable of apoptosis [40]. While MG132 affected relative expression of several proteins involved in apoptosis, it is not clear whether such effects would make the oocyte more or less susceptible to pro-apoptotic signals. MG132 decreased amounts of several proteins that exert anti-apoptotic actions including ASNS [41], HSP90B1 [42], PDIA3 [43], and VCP [44]. Another protein decreased by MG132, CDK5, can lead to apoptosis if aberrantly activated [45] and one protein increased by MG132, P4HB, is anti-apoptotic [46].

One protein decreased by MG132, VCP, has been implicated as an oocyte-derived sperm attractant in ascidians [47]. It remains to be determined whether this protein plays a similar role in mammals. In any case, addition of MG132 from 16–22 h of maturation did not affect fertilization or alter the rate of polyspermy.

The dose-response curve for oocytes exposed to MG132 from 16–22 of maturation was unusual. The optimal beneficial effect was achieved with 10 µM and lower or higher concentrations were not generally effective. Similar effects have been seen in mouse, goat and pig oocytes used for somatic cell nuclear transfer [22], [26] as well as for aged mouse oocytes fertilized using intracytoplasmic sperm injection [6]. One possibility is that residual amounts of MG132 in oocytes treated with high concentrations of MG132 interfere with fertilization or subsequent embryonic development. Indeed, functional proteasomes are required for fertilization [48].

One potential use of MG132 is to improve embryo yield from systems of embryo production based on in vitro maturation of oocytes. Results of the embryo transfer experiment reported here indicates that embryos produced from oocytes treated with MG132 from 16–22 h of maturation have the ability to establish pregnancy after transfer to recipients that is generally similar to control embryos. Thus, even though MG132 did rescue some oocytes that might otherwise might not have been fertilized, there was no noticeable decrease in embryo competence for establishment of pregnancy. A larger study with more embryos is needed to verify this observation.

In conclusion, our results confirm previous findings that inhibition of proteasomal activity early in oocyte maturation can block progression through meiosis and provide new information that inhibition of proteasomes late in maturation can improve the competence of the oocyte to cleave and the resultant embryo to develop to the blastocyst stage. Such results imply that aging-like effects on the oocyte mediated by proteasomes at the end of maturation can compromise the function of the oocyte and implies that yield of embryos from in vitro embryo production systems can be improved by appropriately-timed treatment with MG132. Results from the embryo transfer experiment would suggest that embryo yield can be increased without a loss of competence to establish pregnancy after transfer to recipients.

## Supporting Information

Figure S1 **Chromatograms (280 nm detection) for strong cation exchange chromatography of iTRAQ labeled peptides.** Panel A is the chromatogram from analysis of Set 1 where one control and one MG132 sample were analyzed twice to determine technical replication Panel B is the chromatogram from Set 2 in which two biological replicates of each treatment were analyzed. In both analyses, control was labeled with iTRAQ tags 114 and 115 and MG132 with iTRAQ tags 116 and 117. A total of 12 fractions were submitted to analysis using a quadrupole TOF MS/MS system. The area coverage of each fraction is shown in panel C.
(TIF)

File S1 **Results of analysis of the oocyte proteome.** The first tab contains data from all proteins detected while the second tab is a subset of data from proteins that were differentially expressed between MG132 and vehicle. Cells in which there was significant increase in relative expression caused by MG132 are highlighted in orange whereas cells in which there was a decrease in relative expression are highlighted in blue.
(XLSX)

Table S1 **Number of proteins identified at critical false discovery rates (FDR) from two databases.**
(PDF)
