# Supplementary figures and images for: Treatment with the Proteasome Inhibitor MG132 during the End of Oocyte Maturation Improves Oocyte Competence for Development after Fertilization in Cattle
Source: PLoS One. 2012 Nov 7;7(11):e48613. doi: 10.1371/journal.pone.0048613 (PMC3492449; doi:10.1371/journal.pone.0048613)

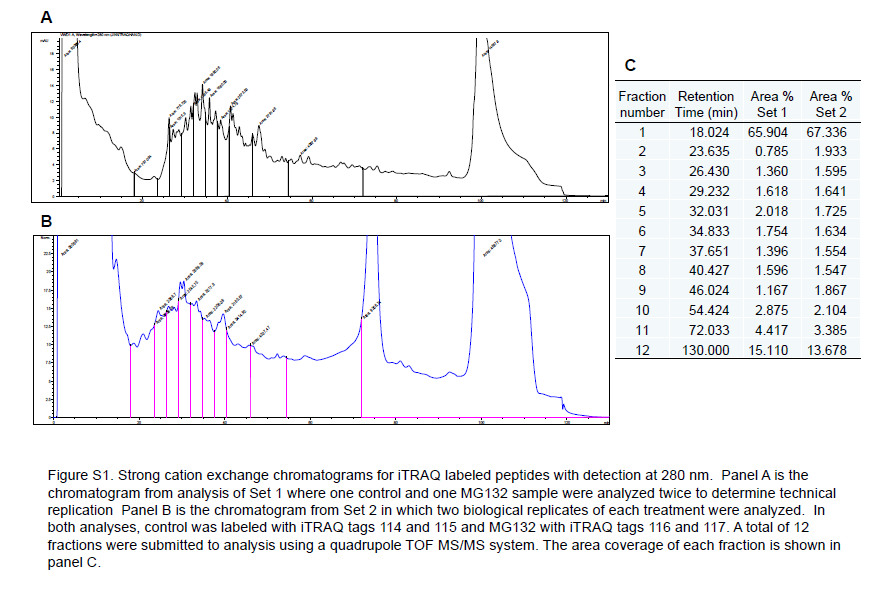

Supplement: Figure S1 — Chromatograms (280 nm detection) for strong cation exchange chromatography of iTRAQ labeled peptides. Panel A is the chromatogram from analysis of Set 1 where one control and one MG132 sample were analyzed twice to determine technical replication Panel B is the chromatogram from Set 2 in which two biological replicates of each treatment were analyzed. In both analyses, control was labeled with iTRAQ tags 114 and 115 and MG132 with iTRAQ tags 116 and 117. A total of 12 fractions were submitted to analysis using a quadrupole TOF MS/MS system. The area coverage of each fraction is shown in panel C. (TIF) [file pone.0048613.s001.tif]
