# Supplementary material for: Treatment with the Proteasome Inhibitor MG132 during the End of Oocyte Maturation Improves Oocyte Competence for Development after Fertilization in Cattle
Source: PLoS One. 2012 Nov 7;7(11):e48613. doi: 10.1371/journal.pone.0048613 (PMC3492449; doi:10.1371/journal.pone.0048613)
Supplement: Table S1 — Number of proteins identified at critical false discovery rates (FDR) from two databases. (PDF) [file pone.0048613.s003.pdf]

Table S1. Number of proteins identified at critical false discovery rates (FDR) from two databases.

FDR from the NCBI database			
Accepted FDR	Local FDR	Global FDR	Global FDR from Fit
0.01%	441	532	534
0.05%	497	587	589
0.10%	515	636	633

FDR from the Uniprot database			
Accepted FDR	Local FDR	Global FDR	Global FDR from Fit
0.01%	447	503	529
0.05%	493	583	591
0.10%	510	643	645
